# Barriers and Facilitators for Older Adults with Serious Mental Illness to Utilizing Medicaid Smartphone Services

**DOI:** 10.1093/geroni/igaa057.3181

**Published:** 2020-12-16

**Authors:** Amanda Myers, Karen Fortuna

**Affiliations:** 1 Rivier University, Hudson, Massachusetts, United States; 2 Dartmouth College, Concord, New Hampshire, United States

## Abstract

As increasingly more older adults in the general population utilize smartphones to access health services, the digital divide between older adults with serious mental illness (SMI) and the general older adult population continues to widen. The purpose of this study was to examine older adult peer support specialists’ and older people with SMI’s perspectives of barriers and facilitators to utilizing Medicaid Safelink smartphone services. Data from two focus groups and five semi-structured interviews from older adult peer support specialists (N=10) and older adults with SMI (N=15) were analyzed using the Consolidated Framework for Implementation Science Research. A mixed methods convergent design integrated quantitative with qualitative data. Older adults with SMI (N= 15) had a mean age of 55 years and were mainly women (70%) and White (100%). Certified peer specialists (N= 10) had a mean age of 52 years (age range 45-67) and were mainly female (75%), 66% identified as White, and 33% identified as African American. Four themes that were identified across different aspects of barriers included technology knowledge, technology adoption, design features (i.e., smartphone size, option to increase font sizes, multi-modal capacity, navigational architecture, 508 compliance), and Safelink policies and procedures. Facilitators included free and continuous services, access to technical support, and smartphone capabilities to enable healthcare communications and facilitate the delivery of services. Improving upon the themes identified as barriers to utilizing Safelink may promote a continuum of care for older adults with SMI, closing the gap of services that occurs between in-person therapy and other interventions.

